# Accumulation of 4-Deoxy-7-hydroxytrichothecenes, but Not 4,7-Dihydroxytrichothecenes, in Axenic Culture of a Transgenic Nivalenol Chemotype Expressing the NX-Type *FgTri1* Gene

**DOI:** 10.3390/ijms222111428

**Published:** 2021-10-22

**Authors:** Kazuyuki Maeda, Yuichi Nakajima, Yoshiaki Koizumi, Naoko Takahashi-Ando, Makoto Kimura, Shuichi Ohsato

**Affiliations:** 1 Faculty of Agriculture, Meiji University, 1-1-1 Higashi-Mita, Tama-ku, Kawasaki 214-8571, Japan; ohsato@meiji.ac.jp; 2 Organization for the Strategic Coordination of Research and Intellectual Properties, Meiji University, 1-1-1 Higashi-Mita, Tama-ku, Kawasaki 214-8571, Japan; 3 Graduate School of Bioagricultural Sciences, Nagoya University, Furo-cho, Chikusa, Nagoya 464-8601, Japan; ynakajima.gm@gmail.com (Y.N.); mkimura@agr.nagoya-u.ac.jp (M.K.); 4 Graduate School of Science and Engineering, Toyo University, Kujirai 2100, Kawagoe 350-0815, Japan; s36d02000045@toyo.jp (Y.K.); ando_n@toyo.jp (N.T.-A.)

**Keywords:** cytochrome P450 monooxygenase gene, evolution, *Fusarium* mycotoxin, NX-type trichothecenes, trichothecene chemotype

## Abstract

*Fusarium graminearum* species complex produces type B trichothecenes oxygenated at C-7. In axenic liquid culture, *F. graminearum* mainly accumulates one of the three types of trichothecenes, namely 3-acetyldeoxyinvalenol, 15-acetyldeoxyinvalenol, or mixtures of 4,15-diacetylnivalenol/4-acetylnivalenol, depending on each strain’s genetic background. The acetyl groups of these trichothecenes are slowly deacetylated to give deoxynivalenol (DON) or nivalenol (NIV) on solid medium culture. Due to the evolution of *F. graminearum FgTri1*, encoding a cytochrome P450 monooxygenase responsible for hydroxylation at both C-7 and C-8, new derivatives of DON, designated as NX-type trichothecenes, have recently emerged. To assess the risks of emergence of new NX-type trichothecenes, we examined the effects of replacing *FgTri1* in the three chemotypes with *FgTri1__NX chemotype_*, which encodes a cytochrome P450 monooxygenase that can only hydroxylate C-7 of trichothecenes. Similar to the transgenic DON chemotypes, the transgenic NIV chemotype strain accumulated NX-type 4-deoxytrichothecenes in axenic liquid culture. C-4 oxygenated trichothecenes were marginal, despite the presence of a functional *FgTri13* encoding a C-4 hydroxylase. At present, outcrossing of the currently occurring NX chemotype with NIV chemotype strains of *F. graminearum* in the natural environment likely will not yield a new strain that produces a C-4 oxygenated NX-type trichothecene.

## 1. Introduction

Trichothecenes are sesquiterpenoid mycotoxins produced by *Fusarium* and fungi from other genera. The presence of a C-8 ketone at the 12,13-epoxytrichothec-9-ene skeleton differentiates type B trichothecenes from type A trichothecenes [1,2]. Among the type B trichothecene producers, the *Fusarium graminearum* species complex [3], the main causal agent of Fusarium head blight in wheat and barley, poses potential threats to the safety of agricultural products. It contaminates infected grains with type B 7-hydroxytrichothecenes, including 4-deoxynivalenol (DON), nivalenol (NIV), and their acetylated derivatives, including 3-acetyl-4-deoxynivalenol (3-ADON), 15-acetyl-4-deoxynivalenol (15-ADON), and 4-acetylnivalenol (4-ANIV; also called fusarenon X).

*F. graminearum* is largely divided into two chemotypes: an NIV chemotype, which has a functional C-4 hydroxylase encoded by a cytochrome P450 monooxygenase gene, *FgTri13*, and a DON chemotype with a dysfunctional copy of *FgTri13* [4,5]. On the basis of the name of the last precursor that accumulates in the rate-limiting deacetylation step to DON, the DON chemotype is further divided into 3-ADON and 15-ADON chemotypes. In axenic liquid culture, 3-ADON and 15-ADON chemotype strains mainly accumulate 3-ADON and 15-ADON, respectively [6,7]. Similarly, an NIV chemotype strain accumulates acetylated derivatives of NIV, namely mixtures of 4-ANIV and 4,15-diacetylnivalenol (4,15-diANIV) [8]. The acetyl groups of these trichothecenes are slowly deacetylated to give DON and NIV when *F. graminearum* strains are cultured on solid medium.

In 2014, the emergence of *F. graminearum* that synthesizes a new trichothecene unable to be detected by the validated LC-MS/MS method for known trichothecenes was reported in North America. The new trichothecene, 7-hydroxy-15-deacetylcalonectrin (7-H-15-deCAL; designated NX-2), lacked a C-8 ketone of 3-ADON [9]. Because they lack a C-8 ketone conjugated to the 9,10-double bond, NX-type trichothecenes cannot be detected by UV absorbance in high-performance liquid chromatography-based methods [10]. An NX-2 chemotype is hypothesized to have evolved from the 3-ADON chemotype because the structural organization of the trichothecene core gene cluster is the same as that of the 3-ADON chemotype [11]. Thus, the multifunctional cytochrome P450 monooxygenase *FgTri1*, a trichothecene noncore cluster gene responsible for hydroxylation at both C-7 and C-8, may have evolved into a monofunctional *FgTri1__NX chemotype_* gene by losing its C-8 hydroxylation function [10].

*Fusarium* strains that produce NX-type trichothecenes oxygenated at C-4 are not known to date. Outcrossing the known NX chemotype, which produces C-8 unoxygenated 4-deoxytrichothecenes, with a known NIV chemotype that has a functional copy of *FgTri13* may lead to the formation of a new NX-type trichothecene oxygenated at C-4 in the natural environment. To assess the possibility of the emergence of such a new chemotype, we genetically engineered known DON and NIV chemotype strains by exchanging their *FgTri1* with *FgTri1__NX chemotype_*.

## 2. Results

### 2.1. Strong Expression of FgTri1__NX chemotype_ in a FgTri1 Disruption Mutant (ΔFgtri1) of Each Chemotype

**Figure 1:**
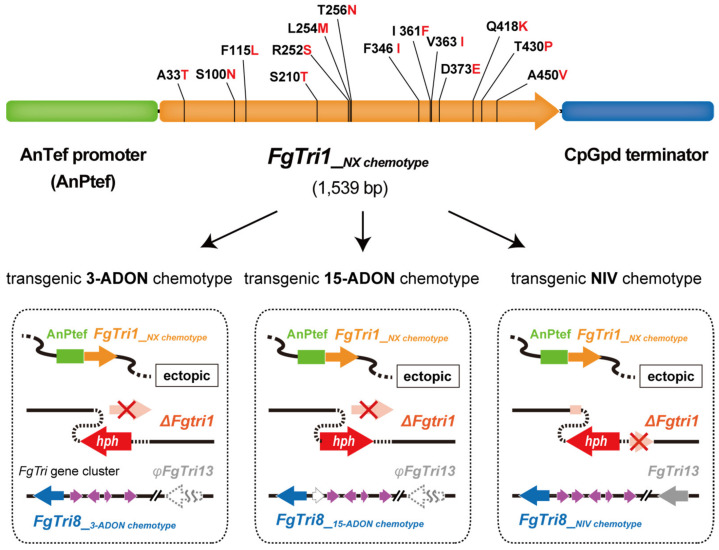
Generation of transgenic *Fusarium* strains expressing the C-7 hydroxylase gene, *FgTri1__NX chemotype_*, with different chemotypic backgrounds. The limited hydroxylation function at C-7 results strictly from 14 amino acid substitutions in the coding region of the evolved *FgTri1__NX chemotype_*. AnTef: *Aspergillus nidulans* translation elongation factor; CpGpd: *Cryphonectria parasitica* glyceraldehyde 3-phosphate dehydrogenase [13].

The sources of the 3-ADON, 15-ADON, and NIV chemotype strains used and the procedures used to generate the transgenic strains are described in detail in the Supplementary Materials. A *FgTri1* disruption mutant (*ΔFgtri1*) of each DON chemotype was generated by double-crossover homologous recombination (Figure S1). Strain NBRC 113176 generated in our previous study [12] was used as a *ΔFgtri1* strain of NIV chemotype. A synthetic *FgTri1__NX chemotype_* gene (1539 bp) was designed such that the 14 amino acids previously identified as being specific to the NX-2 chemotype [10] were conserved (Figure S2). *FgTri1__NX chemotype_* was ectopically expressed in a *ΔFgtri1* mutant of each chemotype under the strong promoter (AnPtef) of the *Aspergillus nidulans* translation elongation factor gene [13] (Figure 1). As expected, the expression levels of *FgTri1__NX chemotype_* transcribed from the AnPtef promoter were much higher than those of the wild-type strain in each chemotype (Figure S3). The resulting transgenic strains, *ΔFgtri1*/*FgTri1__NX chemotype_*, proved to be useful for investigating the function of the *FgTri1__NX chemotype_* gene and trichothecene products.

### 2.2. Time Course of Accumulation of NX-Type Trichothecenes in Axenic Liquid Culture of 3-ADON and 15-ADON Chemotype Strains with Strong Expression of FgTri1__NX chemotype_

**Figure 2:**
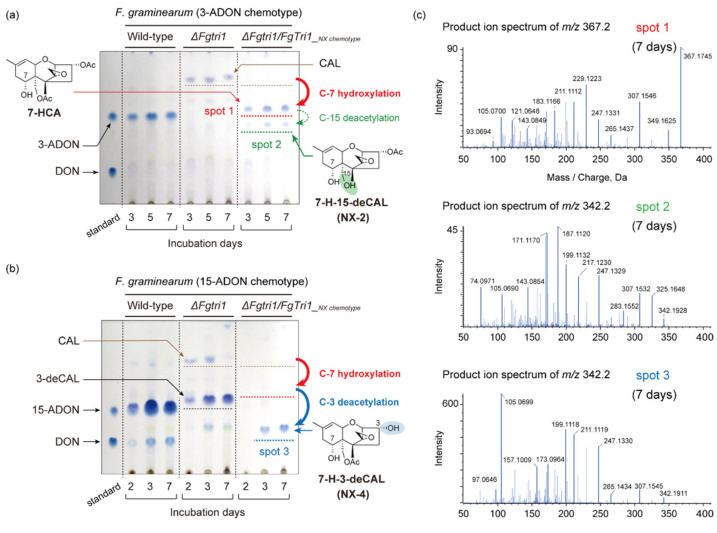
Trichothecene profiles of transgenic 3-ADON and 15-ADON chemotype strains, in which *FgTri1* was replaced with *FgTri1__NX chemotype_* (*F. graminearum ΔFgtri1/FgTri1__NX chemotype_*). (**a**) Time course of trichothecene accumulation of the *ΔFgtri1* and *ΔFgtri1*/*FgTri1__NX chemotype_* mutants of the 3-ADON chemotype visualized by TLC using the 4-(4-nitrobenzyl)pyridine (NBP)/tetraethylene pentamine (TEPA) method [12]. (**b**) Time course that is essentially the same as (**a**), but of mutants of the 15-ADON chemotype. The toxin profiles of each chemotype (wild-type) are also shown for comparison. The structures were confirmed by LC-MS/MS analysis (**c**). MS/MS spectra of trichothecenes included in TLC spots 1, 2, and 3 (red, green, and blue dotted lines). The spectra of *m*/*z* 367.2, *m*/*z* 342.2, and *m*/*z* 342.2, which correspond to [7-HCA + H]^+^ (*m*/*z* 367.1751), [7-H-15-deCAL (NX-2) + NH_4_]^+^ (*m*/*z* 342.1911), and [7-H-3-deCAL (NX-4) + NH_4_]^+^ (*m*/*z* 342.1911), are shown. Dense blue spots on the lanes of the *ΔFgtri1* mutant include 3-deacetylcalonectrin (3-deCAL), a shunt metabolite of CAL [12].

First, we sought to determine whether the transgenic NX-type trichothecene-producing strains could be further divided into NX-2 and NX-4 chemotypes by introducing the aforementioned 14 amino acid mutations into the coding region of *FgTri1* in the 3-ADON and 15-ADON chemotypes, respectively. Analysis of trichothecene metabolite accumulation by thin-layer chromatography (TLC) revealed that the trichothecene profiles of the *ΔFgtri1*/*FgTri1__NX chemotype_* strains differed from those of the wild-type and *ΔFgtri1* strains (Figure 2a,b). In addition, the time course of accumulation of trichothecenes was quite different between the *ΔFgtri1*/*FgTri1__NX chemotype_* strains generated from the 3-ADON and 15-ADON chemotypes. Spot 1, detected from a culture of the transgenic strain of the 3-ADON chemotype, behaved like a trichothecene intermediate at the rate-limiting step to yield spot 2 (Figure 2a). However, the reaction was so slow that spot 1 remained even after 7 days of incubation. In contrast, no precursors were detected in a culture of the transgenic strain derived from the 15-ADON chemotype; spot 3 promptly appeared on day 3, without accumulation of any earlier intermediates (Figure 2b). The trichothecenes included in spots 1–3 were thought to have been synthesized via calonectrin (CAL), an intermediate that accumulates in the *ΔFgtri1* mutant [12,14], by the function of *FgTri1__NX chemotype_* (Figure 2).

The engineered biosynthetic pathways of the transgenic NX chemotypes originating from the 3-ADON and 15-ADON chemotypes are considered to proceed via 7-hydroxycalonectrin (7-HCA) to each NX-type trichothecene; the metabolite is assumed to be deacetylated to yield 7-hydroxy-15-deacetylcalonectrin (7-H-15-deCAL: NX-2) and 7-hydroxy-3-deacetylcalonectrin (7-H-3-deCAL: NX-4) [10] (Figure 2). To confirm the trichothecene metabolites (spots 1–3), culture extracts of the *ΔFgtri1*/*FgTri1__NX chemotype_* strains were analyzed. In liquid chromatography–tandem mass spectrometry (LC-MS/MS) analysis of the metabolites of the transgenic 3-ADON chemotype, the extracted ion chromatograms (XIC) of *m*/*z* 367.175 ± 0.025 (retention time 3.68 min), corresponding to [7-HCA + H]^+^ (*m*/*z* 367.1751), showed a peak, the MS/MS spectrum of which was superimposable to that of 7-HCA included in our in-house trichothecene MS/MS library database [12,15]. Similarly, the MS/MS spectrum of *m*/*z* 342.191 ± 0.025 (retention time 3.13 min) corresponded well to that of [7-H-15-deCAL (NX-2) + NH_4_]^+^ (*m*/*z* 342.1911). These results demonstrate that the trichothecenes in spots 1 and 2 were 7-HCA and NX-2, respectively (Figure 2c). In LC-MS/MS analysis of the metabolites of the transgenic 15-ADON chemotype, the MS/MS fragmentation pattern of a peak in XIC of *m*/*z* 342.191 ± 0.025 (retention time 2.81 min) was identical to that of [7-H-3-deCAL (NX-4) + NH_4_]^+^ (*m*/*z* 342.1911) in the database, confirming that spot 3 was NX-4 (Figure 2c).

### 2.3. FgTri1__NX chemotype_ Overexpressing Strain of NIV Chemotype Accumulates 4-Deoxy-7-hydroxytrichothcenes, but Not 4,7-Dihydroxytrichothecenes

**Figure 3:**
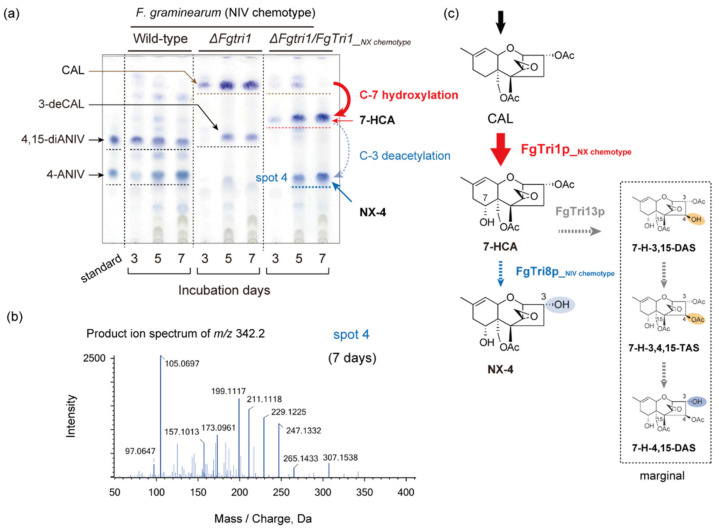
Accumulation of 4-deoxytrichothecenes in a culture of the transgenic NIV chemotype strain of *F. graminearum ΔFgtri1*/*FgTri1__NX chemotype_*. (**a**) Time course of trichothecene accumulation of the wild-type and mutant strains visualized on a TLC plate. Red and blue (spot 4) dotted lines indicate trichothecenes detected in cultures of the *ΔFgtri1*/*FgTri1__NX chemotype_* strain. They were not produced by the wild-type and *ΔFgtri1* strains. The structures were confirmed by LC-MS/MS analysis. Blue spots with an *R*_f_ value lower than that of CAL on the lanes of the *ΔFgtri1* mutant include 3-deCAL [12]. (**b**) MS/MS spectrum of *m*/*z* 342.2, corresponding to [NX-4 + NH_4_]^+^ (*m*/*z* 342.1911). A culture extract of the *ΔFgtri1*/*FgTri1__NX chemotype_* strain, which produced TLC spot 4, was analyzed. (**c**) An engineered biosynthetic pathway of the *ΔFgtri1*/*FgTri1__NX chemotype_* strain. The production of 7-H-4,15-DAS [16], an NX-type C-4 oxygenated trichothecene, was extremely limited. 7-H-3,15-DAS: 7-hydroxy-3,15-diacetoxyscirpenol; 7-H-3,4,15-TAS: 7-hydroxy-3,4,15-triacetoxyscirpenol.

Next, we focused on the possibility of the emergence of a new NX-type trichothecene oxygenated at C-4 (Figure 3). Time-course metabolite profiles of the *ΔFgtri1*/*FgTri1__NX chemotype_* NIV chemotype strain by TLC revealed the appearance of a trichothecene spot 4, whose retention factor (*R*_f_) value was identical to that of spot 3, on day 5 (Figure 3a). However, in contrast to the transgenic 15-ADON chemotype, 7-HCA accumulation was observed for this strain. To confirm the structures of the trichothecenes, a culture extract of the transgenic strain after 7 days of culture was analyzed by LC-MS/MS. In XIC chromatograms of *m*/*z* 367.175 ± 0.025 and *m*/*z* 342.191 ± 0.025, major peaks corresponding to [7-HCA + H]^+^ (*m*/*z* 367.1751) and [7-H-3-deCAL (NX-4) + NH_4_]^+^ (*m*/*z* 342.1911), respectively, were detected at 3.67 min and 2.81 min. The fragmentation patterns of the peaks were similar to those of the transgenic DON chemotypes (Figure 2c), indicating that the metabolites in the culture extract were indeed 7-HCA and NX-4 (spot 4) (Figure 3b). Thus, the final toxin product of the transgenic strain in axenic liquid culture, spot 4, was the same product included in spot 3 of the transgenic 15-ADON chemotype. Despite our efforts to identify the C-4 oxygenated NX-type trichothecenes, minor C-4 oxygenated trichothecenes, including the previously identified 7-hydroxy-4,15-diacetoxyscirpenol (7-H-4,15-DAS) [16], were barely detected, or detected in marginal quantities, in a culture of the transgenic *ΔFgtri1*/*FgTri1__NX chemotype_* strain.

## 3. Discussion

We used the three known type B trichothecene chemotypes as hosts to investigate the effect of the expression of a recently evolved trichothecene pathway gene, *FgTri1__NX chemotype_*. Although the production of NX-4 by an engineered 15-ADON chemotype strain has been previously reported [10], the efficiency of 3-*O*- and 15-*O*-deacetylations of 7-HCA in an axenic culture of *F. graminearum* was not compared. The time-course experiment (Figure 2b) revealed that conversion of intermediate 7-HCA to NX-4 was so rapid that 7-HCA was not detected from the culture of the transgenic 15-ADON chemotype. In contrast, quite a few amounts of 7-HCA, showing a larger blue spot on TLC than that of NX-2, accumulated in the culture of the transgenic 3-ADON chemotype (Figure 2a). The very slow formation of NX-2, but not NX-4, by the transgenic 3-ADON and 15-ADON chemotypes suggested that the 15-*O*-acetyl group of 7-HCA is biologically more stable than the 3-*O*-acetyl group. This may be attributed to the function of *FgTri8*, a trichothecene deacetylase gene responsible for 3-ADON/15-ADON chemotype differentiation. FgTri8p__15-ADON chemotype_ C-3 deacetylase of the 15-ADON chemotype appears to accept various trichothecenes as substrates, as opposed to FgTri8p__3-ADON chemotype_ C-15 deacetylase of the 3-ADON chemotype [17]. In addition to the difference in the toxin formation kinetics between the two chemotypes, the present study unambiguously demonstrated that C-4 oxygenated trichothecenes marginally accumulated in a culture of the NIV chemotype harboring the *FgTri1__NX chemotype_* gene. The result suggests that CAL and 7-HCA were poor substrates of FgTri13p, supporting our previous finding of the specificity of the FgTri13p enzyme towards trichothecene intermediates [12]. These findings shed light on the biosynthetic pathway of NIV after CAL in *F. graminearum*. Thus, outcrossing of the currently occurring NX-2 chemotype [3,9,10,18,19,20] and NIV chemotype strains of *F. graminearum* in the natural environment likely will not yield a new strain that produces a C-4 oxygenated NX-type trichothecene. The *Tri13* genes are widely distributed among trichothecene-producing FIESC (*F. incarnatum-equiseti* species complex) and Sambucinum species complex [21]. A previous study reported that the amino acid sequence similarity of Tri13p ranges from 76% to 100% [22]. The sequence diversity indicates that there is a possibility for Tri13p to hydroxylate the C-4 unoxygenated NX-type trichothecene in other *Fusarium* species. In fact, an isolate of *Fusarium equiseti*, a member of FIESC, was reported to accumulate a small amount of 7-H-4,15-DAS in axenic culture [16]. The mechanisms underlying the diversity of trichothecene side-chain variations need to be elucidated by analyzing the functions of *Tri1* and *Tri13* of various trichothecene-producing *Fusarium* species in terms of the function and substrate specificity of the biosynthetic monooxygenases they encode.

## 4. Materials and Methods

### 4.1. Strains

The trichothecene-producing strains of the *F. graminearum* species complex used in this study were obtained from the Ministry of Agriculture, Forestry and Fisheries (MAFF) and the Japan Collection of Microorganisms (JCM), including MAFF 111233 (*Fusarium asiaticum*), an NIV chemotype; MAFF 101551 (*F. graminearum* sensu stricto), a 3-ADON chemotype; JCM 9873 (*F. graminearum* sensu stricto); and a 15-ADON chemotype. Strain NBRC (NITE Biological Resource Center) 113176 (*ΔFgtri1*) is a previously described strain derived from MAFF 111233 [12]. The parental and transgenic strains were maintained on V8 juice agar medium (20% Campbell’s V8 juice, 0.3% CaCO_3_, 2% agar) at 25 °C with or without hygromycin B (300 µg/mL) and/or G418 (100 µg/mL).

### 4.2. Production of Trichothecenes

For trichothecene induction, mycelial plugs of wild-type and transformants of the NIV chemotype were inoculated onto boiled rice flour (RF) liquid medium (5% rice flour, 3% sucrose, 0.1% yeast extract) and cultured as described previously [17]. DON chemotype strains were cultured on carboxymethyl cellulose liquid medium (1.5% carboxymethyl cellulose sodium salt, 0.1% NH_4_NO_3_, 0.1% KH_2_PO_4_, 0.1% yeast extract, 0.05% MgSO_4_·7H_2_O) to induce conidiation for trichothecene production. The conidial suspension was inoculated onto YG preculture medium (0.5% yeast extract, 2% glucose) at 25 °C for 16 h under reciprocal shaking (135 rpm). For trichothecene induction, 300 µL of germinated conidia was transferred to 30 mL of medium in a 100 mL Erlenmeyer flask under gyratory shaking (135 rpm) at 25 °C. YS_60 medium (6% sucrose, 0.1% yeast extract) and 30 mL of Gln synthetic medium [23,24] were used for induction of the 3-ADON and 15-ADON chemotypes, respectively.

### 4.3. Reagents

A TLC plate (Glass TLC plate, silica gel coated with fluorescent indicator F_254_), polyethylene glycol 4000, and hyper-grade acetonitrile for liquid chromatography–mass spectrometry (LC-MS) were obtained from Merck KGaA (Darmstadt, Germany). BD Bacto yeast extract (Lot No. 1186275), hygromycin B, G418 sulfate, NBP, TEPA, TLC solvent, and the other chemicals were purchased from FUJIFILM Wako Pure Chemical (Osaka, Japan).

### 4.4. Construction of Transformation Vectors

*FgTri1* gene disruption vectors were constructed by replacing the coding region of *FgTri1* with a hygromycin B phosphotransferase gene (*hph*) cassette by using different methods. For the construction of pH3ΔFgTri1-hph (Figure S1a), an *hph* cassette without *trpC* terminator from pCSN43 [25] and the flanking regions of *FgTri1* concatenated with 16-bp overlapping sequences were PCR-amplified using the primers listed in Table S1 and assembled directionally into a *Sma*I-linearized pUC19 vector using a NEBuilder HiFi DNA Assembly Master Mix (New England Biolabs, Ipswich, MA, USA) according to the instruction manual. pJCMΔFgTri1-hph was constructed by inverse PCR as described previously (Figure S1b) [12,26]. A trichothecene C-7 hydroxylase-encoding *FgTri1__NX chemotype_* gene was synthetized by Eurofins Genomics (K.K., Tokyo, Japan) and supplied in a cloning plasmid, pEX-A2J1. The *FgTri1__NX chemotype_* expression vector was constructed as follows: the plasmid was digested with *Pac*I and *Asc*I restriction endonucleases, and the synthetic gene, cloned between the *Pac*I and *Asc*I recognition sites, was excised from the vector backbone. An overexpression vector, pAnTef-neo [17], which contains the neomycin phosphotransferase gene (*neo*) cassette, was digested with the same enzymes and ligated with the synthetic *FgTri1__NX chemotype_* gene using a Ligation-Convenience Kit (Nippon Gene, CO., LTD., Tokyo, Japan) to construct the destination vector, pAnTef-*FgTri1__NX chemotype_*-neo (Figure S2).

### 4.5. Generation of Transformants

Genetic transformation of *F. graminearum* species was performed according to a previous report [27], with slight modification. In brief, constructed vectors were linearized with *Hin*dIII (for pH3ΔFgTri1-hph), *Nhe*I (for pJCMΔFgTri1-hph), or *Xba*I (for pAnTef-*FgTri1__NX chemotype_*-neo) and purified using a GEL/PCR Purification Mini Kit (Favorgen Biotech, Ping-Tung, Taiwan). Thirty micrograms of purified DNA was mixed with 500 µL of protoplast suspension (1 × 10^8^ cells/mL) and incubated at room temperature for 20 min. Then, the suspension was incubated at 42 °C for 15 min, after which two volumes of PEG/CaCl_2_ solution (60% polyethylene glycol 4000, 10 mM Tris-HCl, 50 mM CaCl_2_, pH 8.0) were added in a stepwise manner. Following a 30-min incubation, the protoplasts were washed once with 30 mL of STC50 buffer (1.2 M sorbitol, 10 mM Tris-HCl, 50 mM CaCl_2_, pH 7.5) and suspended in 1.5 mL of YG1/2SuC (0.5% yeast extract, 2% glucose, 0.8 M sucrose, 25 mM CaCl_2_). After incubation at 25 °C under gentle reciprocal shaking (80–90 rpm) for 5 h, the regenerated protoplast suspension was spread onto Down agar (0.8 M sucrose, 0.5% yeast extract, 1.2% agar) containing appropriate concentrations of selection markers (30 µg/mL hygromycin B, 10 µg/mL G418). The agar medium was overlaid with top agar (1 M sorbitol, 1% agarose) containing the same antibiotics and allowed to solidify. Three to four days after cultivation, antibiotic-resistant colonies were transferred to potato dextrose agar (Eiken Chemical, Tokyo, Japan) with higher concentrations of the selection markers (150 µg/mL hygromycin B, 50 µg/mL G418) and further selected. Candidate transformants harboring *FgTri1* gene disruption mutants were further investigated by PCR as described in Figure S1c,d.

### 4.6. Nucleic Acid Manipulations

Genomic DNA was extracted from fungal tissues cultured in YG medium with or without selection markers for 3 days, using a Nucleon PhytoPure kit (GE Healthcare Bioscience, Tokyo, Japan) according to the instruction manual. RNA extraction and cDNA synthesis (from 1 µg of total RNA) were performed as previously reported [28]. The cDNA was used as a template for RT-PCR. In the RT-PCR analysis, *FgTri1* and the ubiquitin-conjugating enzyme gene (*Ubc*; as a positive control) were amplified using the primer sets listed in Table S1 and detected under UV (Figure S3).

### 4.7. Trichothecene Analysis

Trichothecene cultures were collected at appropriate time points during cultivation for up to 7 days. Trichothecene was extracted from the culture supernatant by adding an equal volume of ethyl acetate. Dried extract was applied onto a TLC plate, developed (ethyl acetate: toluene = 3:1), and detected as blue-purple spots based on the NBP/TEPA color reaction [12]. LC-MS/MS analysis for trichothecene confirmation was carried out using an Eksigent ekspert ultraLC 100-XL system (Dublin, CA, USA) equipped with a C18 reverse-phase column (PEGASIL ODS SP100-3; 2φ × 100 mm, Senshu Scientific Co., Ltd., Tokyo, Japan), as previously described [12,17].

## Data Availability

The data underlying this article are available in the article and in its online Supplementary Materials.

## Supplementary Materials

The following are available online at https://www.mdpi.com/article/10.3390/ijms222111428/s1.

## Abbreviations

3-ADON	3-Acetyl-4-deoxynivalenol
3-deCAL	3-Deacetylcalonectrin
4,15-diANIV	4,15-Diacetylnivalenol
4-ANIV	4-Acetylnivalenol
7-H-15-deCAL	7-Hydroxy-15-deacetylcalonectrin
7-H-3-deCAL	7-Hydroxy-3-deacetylcalonectrin
7-H-3,15-DAS	7-Hydroxy-3,15-diacetoxyscirpenol
7-H-3,4,15-TAS	7-Hydroxy-3,4,15-triacetoxyscirpenol
7-H-4,15-DAS	7-Hydroxy-4,15-diacetoxyscirpenol
7-HCA	7-Hydroxycalonectrin
15-ADON	15-Acetyl-4-deoxynivalenol
AnPtef	*Aspergillus nidulans* translation elongation factor promoter
CAL	Calonectrin
DON	4-Deoxynivalenol
FIESC	*Fusarium incarnatum-equiseti* species complex
*hph*	Hygromycin B phosphotransferase gene
JCM	Japan Collection of Microorganisms
LC-MS	Liquid chromatography–mass spectrometry
LC-MS/MS	Liquid chromatography–tandem mass spectrometry
MAFF	Ministry of Agriculture, Forestry and Fisheries
NBP	4-(4-Nitrobenzyl)pyridine
NBRC	NITE Biological Resource Center
neo	Neomycin phosphotransferase gene
NIV	Nivalenol
*R*_f_	Retention factor
RF	Rice flour
TEPA	Tetraethylene pentamine
TLC	Thin-layer chromatography
Ubc	Ubiquitin-conjugating enzyme
XIC	Extracted ion chromatograms
