# Supplementary material for: Accumulation of 4-Deoxy-7-hydroxytrichothecenes, but Not 4,7-Dihydroxytrichothecenes, in Axenic Culture of a Transgenic Nivalenol Chemotype Expressing the NX-Type FgTri1 Gene
Source: Int J Mol Sci. 2021 Oct 22;22(21):11428. doi: 10.3390/ijms222111428 (PMC8583793; doi:10.3390/ijms222111428)
Supplement: Supplementary file 1 [file ijms-22-11428-s001.zip › ijms-1420491-supplementary.pdf]

Table S1. T Primers for used in this study.

No.	Primer	Sequence (5′–3′) [1]	Description
#01	PtrpC-Fw	CCCTCGACAGAAGATGATATTGAA	Primer for construction of pH3ΔFgTri1-hph
#02	hph-Rev	ACCCTATTCCTTTGCCCTCGGACG	Primer for construction of pH3ΔFgTri1-hph
#03	delta_Fg_Tri1_U1	CCAGTGAATTCGAGCTTCTATGAGTCTGTGGGGTGGA	Primer for construction of pH3ΔFgTri1-hph
#04	delta_Fg_Tri1_U2	AATAGGGTACCGAGCTGACAGCGAAATGGTCTGTCAA	Primer for construction of pH3ΔFgTri1-hph
#05	delta_Fg_Tri1_D1	CATCTTCTGTCGAGGGGTAGGAGGACGTCACAGTCTT	Primer for construction of pH3ΔFgTri1-hph
#06	delta_Fg_Tri1_D2	CAGGTCGACTCTAGAGCCAAGAGTAAAAGACGCATCC	Primer for construction of pH3ΔFgTri1-hph
#07	FgTri1_Long_Fw	CCATTTTTGTGAGTAGGCCTC	Primer for screening of *FgTri1* gene disruption mutant
#08	FgTri1_Long_Rev	TCTTCGGTTGTTGTGGTCGTT	Primer for screening of *FgTri1* gene disruption mutant
#09	Specific_on_hph	CCGTCTGGACCGATGGCTGTG	Primer for screening of *FgTri1* gene disruption mutant
#10	Specific_on_PtrpC	CAAATTTTGTGATCCGCCTGG	Primer for screening of *FgTri1* gene disruption mutant
#11	JCMdTri1-01F_NheI	ACGCTAGCACATCATGCCAACAGTTAGTT	Inward primer for construction of pJCMΔFgTri1-hph
#12	JCMdTri1-02R_NheI	ACGCTAGCCGTTGGCTCTTTTCAAGTGTC	Inward primer for construction of pJCMΔFgTri1-hph
#13	JCMdTri1-03F_NotI	AAGCGGCCGCTTTGACTACAACGACGTTTGC	Outward primer for construction of pJCMΔFgTri1-hph
#14	JCMdTri1-04R_NotI	AAGCGGCCGCCGACCTACCGAATAATACTGA	Outward primer for construction of pJCMΔFgTri1-hph
#15	JCMdTri1-05F_Long	GTGGTAGGTGGTAAACGAAAA	Primer for screening of *FgTri1* gene disruption mutant
#16	JCMdTri1-06R_Long	GGGAAGAATTAAAGCGTGAGT	Primer for screening of *FgTri1* gene disruption mutant
#17	SacI side	CACTAAAGGGAACAAAAGCTG	Primer for screening of *FgTri1* gene disruption mutant
#18	check on TrpC	TGAATGCTCCGTAACACCCAATA	Primer for screening of *FgTri1* gene disruption mutant
#19	MFTri1_Fw	ATGGCTATCATCAGCAACTTG	Primer for RT-PCR analysis of *FgTri1* toward NIV chemotype
#20	MFTri1_Rev	CTAGTCGTCCTGTACCAATTC	Primer for RT-PCR analysis of *FgTri1* toward NIV chemotype
#21	FgTri1_Fw	ATGGCTCTCATCACCAGTTTG	Primer for RT-PCR analysis of *FgTri1* toward DON chemotypes
#22	FgTri1_Rev	CTAGTCATCCTGTACCAATTC	Primer for RT-PCR analysis of *FgTri1* toward DON chemotypes
#23	Ubc_RT-Fw	CAAGGAGTTGACTGATCTCGG	Primer for RT-PCR analysis of *Ubc*
#24	Ubc_RT-Rev	GCGTACTTTCGAGTCCACTCT	Primer for RT-PCR analysis of *Ubc*

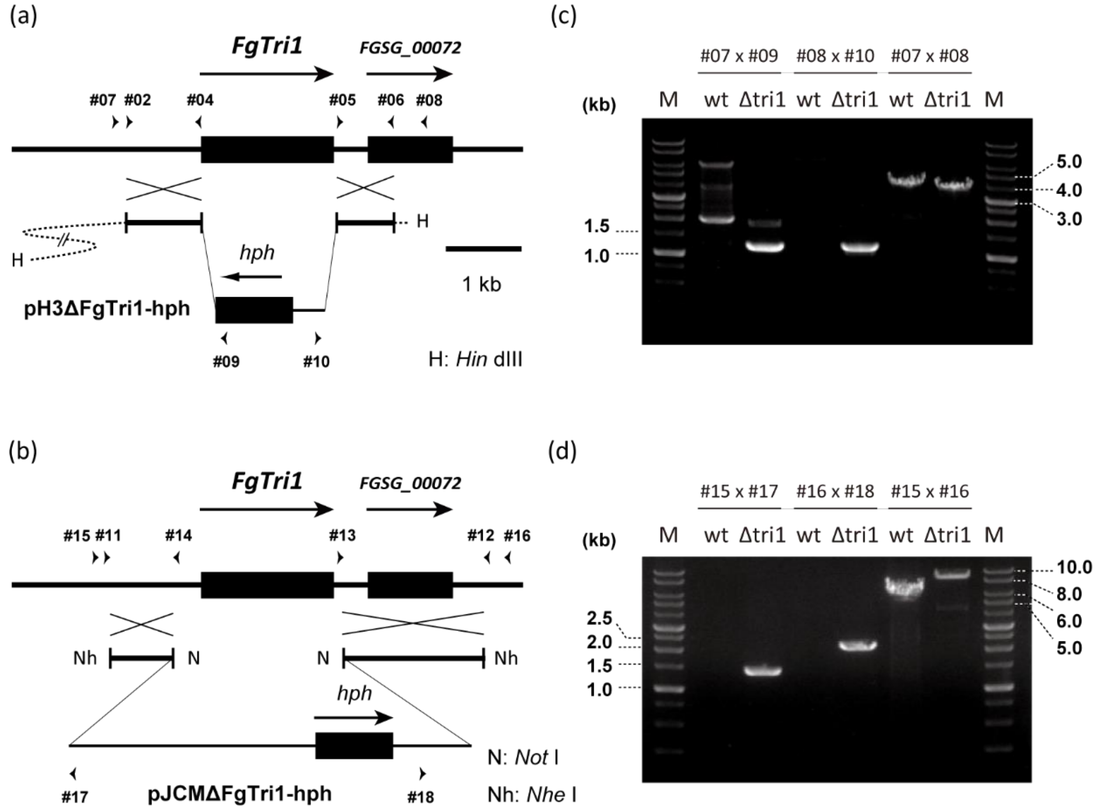

Figure S1. Generation of *FgTri1* gene disruption mutants. Schematic diagram of gene deletion in *F. graminearum* strain MAFF 101551 (**a**) and strain JCM 9873 (**b**). PCR-based confirmation of *FgTri1* gene disruption in MAFF 101551 (**c**) and in JCM 9873 (**d**). Two primer pairs that specifically amplify the junction between *FgTri1* gene-flanking region and gene disruption vector (i.e., #07 × #09 and #08 × #10, or #15 × #17 and #16 × #18) and one primer pair that amplifies the fragment encompassing the deleted region (i.e., #07 × #08, or #15 × #16) were used. Bands characteristic of the *ΔFgtri1* mutation were obtained when PCR was performed with primers #07 and #09 (1.1 kb), #08 and #10 (1.0 kb) and #07 and #08 (3.6 kb) on MAFF 101551, and #15 and #17 (1.4 kb), #16 and #18 (2.0 kb) and #15 and #16 (8.3 kb) on JCM 9873.

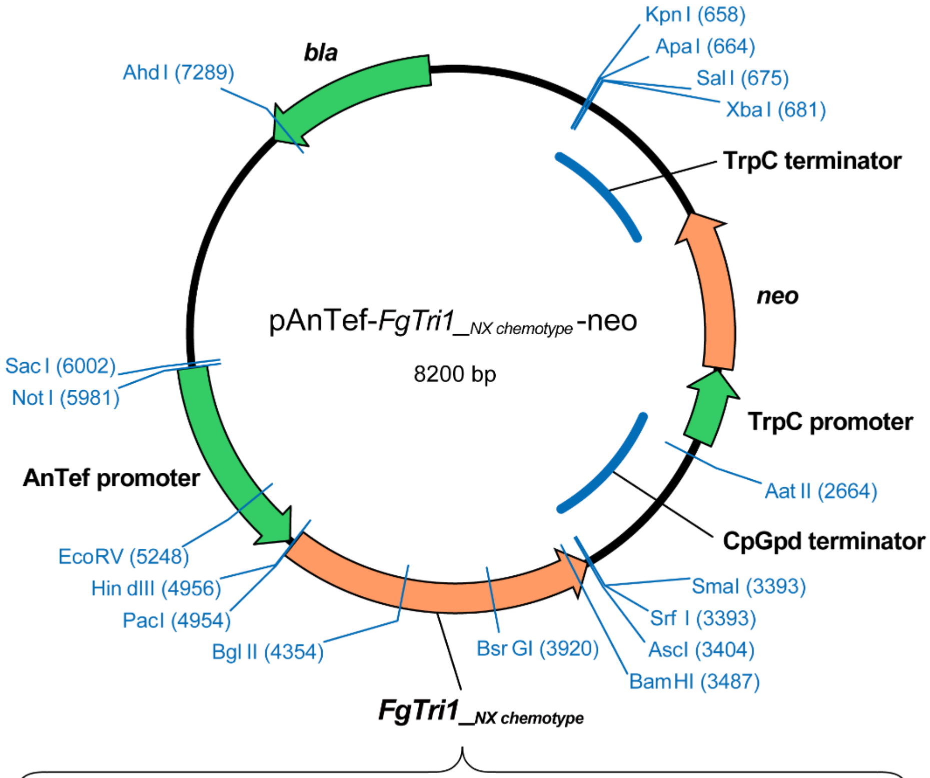

No.	original	substitution	modified	No.	original	substitution	modified
#1	GCC	A 33 T	ACC	#8	TTC	F346I	ATC
#2	AGC	S100N	AAC	#9	ATT	I361F	TTT
#3	TTC	F115L	TTG	#10	GTT	V363I	ATT
#4	TCG	S210T	ACG	#11	GAC	D373E	GAA
#5	CGC	R252S	AGC	#12	CAA	Q418K	AAA
#6	CTA	L254M	ATG	#13	ACC	T430P	CCC
#7	ACT	T256N	AAT	#14	GCC	A450V	GTC

pAnTef-Fg*Tri1*_$_{\mathit{NX\ chemotype}}$-neo

1	CACCTAAATT	GTAAGCGTTA	ATATTTTGTT	AAAATTCGCG	TTAAATTTTT	GTTAAATCAG	CTCATTTTTT	AACCAATAGG	CCGAAATCGG	CAAAATCCCT
	GTGGATTTAA	CATTCGCAAT	TATAAAACAA	TTTTAAGCGC	AATTTAAAAA	CAATTTAGTC	GAGTAAAAAA	TTGGTTATCC	GGCTTTAGCC	GTTTTAGGGA
101	TATAAATCAA	AAGAATAGAC	CGAGATAGGG	TTGAGTGTTG	TTCCAGTTTG	GAACAAGAGT	CCACTATTAA	AGAACGTGGA	CTCCAACGTC	AAAGGGCGAA
	ATATTTAGTT	TTCTTATCTG	GCTCTATCCC	AACTCACAAC	AAGGTCAAAC	CTTGTTCTCA	GGTGATAATT	TCTTGCACCT	GAGGTTGCAG	TTTCCCGCTT
201	AAACCGTCTA	TCAGGGCGAT	GGCCCACTAC	GTGAACCATC	ACCCTAATCA	AGTTTTTTGG	GGTCGAGGTG	CCGTAAAGCA	CTAAATCGGA	ACCCTAAAGG
	TTTGGCAGAT	AGTCCCGCTA	CCGGGTGATG	CACTTGGTAG	TGGGATTAGT	TCAAAAAACC	CCAGCTCCAC	GGCATTTCGT	GATTTAGCCT	TGGGATTTCC
301	GAGCCCCCGA	TTTAGAGCTT	GACGGGGAAA	GCCGGCGAAC	GTGGCGAGAA	AGGAAGGGAA	GAAAGCGAAA	GGAGCGGGCG	CTAGGGCGCT	GGCAAGTGTA
	CTCGGGGGCT	AAATCTCGAA	CTGCCCCTTT	CGGCCGCTTG	CACCGCTCTT	TCCTTCCCTT	CTTTCGCTTT	CCTCGCCCGC	GATCCCGCGA	CCGTTCACAT
401	GCGGTCACGC	TGCGCGTAAC	CACCACACCC	GCCGCGCTTA	ATGCGCCGCT	ACAGGGCGCG	TCCCATTCGC	CATTCAGGCT	GCGCAACTGT	TGGGAAGGGC
	CGCCAGTGCG	ACGCGCATTG	GTGGTGTGGG	CGGCGCGAAT	TACGCGGCGA	TGTCCCGCGC	AGGGTAAGCG	GTAAGTCCGA	CGCGTTGACA	ACCCTTCCCG
501	GATCGGTGCG	GGCCTCTTCG	CTATTACGCC	AGCTGGCGAA	AGGGGGATGT	GCTGCAAGGC	GATTAAGTTG	GGTAACGCCA	GGGTTTTCCC	AGTCACGACG
	CTAGCCACGC	CCGGAGAAGC	GATAATGCGG	TCGACCGCTT	TCCCCCTACA	CGACGTTCCG	CTAATTCAAC	CCATTGCGGT	CCCAAAAGGG	TCAGTGCTGC
						KpnI		SalI	TrpC terminator	
							ApaI		XbaI	
601	TTGTAAAACG	ACGGCCAGTG	AATTGTAATA	CGACTCACTA	TAGGGCGAAT	TGGGTACCGG	GCCCCCCCTC	GAGGTCGACT	CTAGAAAGAA	GGATTACCTC
	AACATTTTGC	TGCCGGTCAC	TTAACATTAT	GCTGAGTGAT	ATCCCGCTTA	ACCCATGGCC	CGGGGGGGAG	CTCCAGCTGA	GATCTTTCTT	CCTAATGGAG
						TrpC terminator				
701	TAAACAAGTG	TACCTGTGCA	TTCTGGGTAA	ACGACTCATA	GGAGAGTTGT	AAAAAAGTTT	CGGCCGGCGT	ATTGGGTGTT	ACGGAGCATT	CACTAGGCAA
	ATTTGTTCAC	ATGGACACGT	AAGACCCATT	TGCTGAGTAT	CCTCTCAACA	TTTTTTCAAA	GCCGGCCGCA	TAACCCACAA	TGCCTCGTAA	GTGATCCGTT
						TrpC terminator				
801	CCATGGTTAC	TATTGTATAC	CCATCTTAGT	AGGAATGATT	TTCGAGGTTT	ATACCTACGA	TGAATGTGTG	TCCTGTAGGC	TTGAGAGTTC	AAGGAAGAAA
	GGTACCAATG	ATAACATATG	GGTAGAATCA	TCCTTACTAA	AAGCTCCAAA	TATGGATGCT	ACTTACACAC	AGGACATCCG	AACTCTCAAG	TTCCTTCTTT
						TrpC terminator				
901	CAGTGCAATT	ATCTTTGCGA	ACCCAGGGGC	TGGTGACGGA	ATTTTCATAG	TCAAGCTATC	AGAGTAAAGA	AGAGGAGCAT	GTCAAAGTAC	AATTAGAGAC
	GTCACGTTAA	TAGAAACGCT	TGGGTCCCCG	ACCACTGCCT	TAAAAGTATC	AGTTCGATAG	TCTCATTTCT	TCTCCTCGTA	CAGTTTCATG	TTAATCTCTG
						TrpC terminator				
1001	AAATATATAG	TCGCGTGGAG	CCAAGAGCGG	ATTCCTCAGT	CTCGTAGGTC	TCTTGACGAC	CGTTGATCTG	CTTGATCTCG	TCTCCCGAAA	ATGAAAATAG
	TTTATATATC	AGCGCACCTC	GGTTCTCGCC	TAAGGAGTCA	GAGCATCCAG	AGAACTGCTG	GCAACTAGAC	GAACTAGAGC	AGAGGGCTTT	TACTTTTATC
						TrpC terminator				
1101	ACTCTGCTAA	GCTATTCTTC	TGCTTCGCCG	GAGCCTGAAG	GGCGTACTAG	GGTTGCGAGG	TCCAATGCAT	TAATGCATTG	CAGATGAGCT	GTATCTGGAA
	TGAGACGATT	CGATAAGAAG	ACGAAGCGGC	CTCGGACTTC	CCGCATGATC	CCAACGCTCC	AGGTTACGTA	ATTACGTAAC	GTCTACTCGA	CATAGACCTT
						TrpC terminator				
1201	GAGGTAAACC	CGAAACGCGT	TTTATTCTTG	TTGACATGGA	GCTATTAAAT	CACTAGAAGG	CACTCTTTGC	TGCTTGGACA	AATGAACGTA	TCTTATCGAG
	CTCCATTTGG	GCTTTGCGCA	AAATAAGAAC	AACTGTACCT	CGATAATTTA	GTGATCTTCC	GTGAGAAACG	ACGAACCTGT	TTACTTGCAT	AGAATAGCTC
						TrpC terminator				
1301	ATCCTGAACA	CCATTTGTCT	CAACTCCGGA	GCTGACATCG	ACACCAACGA	TCTTATATCC	AGATTCGTCA	AGCTGTTTGA	TGATTTCAGT	AACGTTAAGT
	TAGGACTTGT	GGTAAACAGA	GTTGAGGCCT	CGACTGTAGC	TGTGGTTGCT	AGAATATAGG	TCTAAGCAGT	TCGACAAACT	ACTAAAGTCA	TTGCAATTCA
	TrpC terminator									
1401	GGATCGATCC	CCCCCTTCAG	AAGAACTCGT	CAAGAAGGCG	ATAGAAGGCG	ATGCGCTGCG	AATCGGGAGC	GGCGATACCG	TAAAGCACGA	GGAAGCGGTC
	CCTAGCTAGG	GGGGGAAGTC	TTCTTGAGCA	GTTCTTCCGC	TATCTTCCGC	TACGCGACGC	TTAGCCCTCG	CCGCTATGGC	ATTTCGTGCT	CCTTCGCCAG
							neo			
1501	AGCCCATTCG	CCGCCAAGCT	CTTCAGCAAT	ATCACGGGTA	GCCAACGCTA	TGTCCTGATA	GCGGTCCGCC	ACACCCAGCC	GGCCACAGTC	GATGAATCCA
	TCGGGTAAGC	GGCGGTTCGA	GAAGTCGTTA	TAGTGCCCAT	CGGTTGCGAT	ACAGGACTAT	CGCCAGGCGG	TGTGGGTCGG	CCGGTGTCAG	CTACTTAGGT
						neo				
1601	GAAAAGCGGC	CATTTTCCAC	CATGATATTC	GGCAAGCAGG	CATCGCCATG	GGTCACGACG	AGATCCTCGC	CGTCGGGCAT	GCGCGCCTTG	AGCCTGGCGA
	CTTTTCGCCG	GTAAAAGGTG	GTACTATAAG	CCGTTCGTCC	GTAGCGGTAC	CCAGTGCTGC	TCTAGGAGCG	GCAGCCCGTA	CGCGCGGAAC	TCGGACCGCT
						neo				
1701	ACAGTTCGGC	TGGCGCGAGC	CCCTGATGCT	CTTCGTCCAG	ATCATCCTGA	TCGACAAGAC	CGGCTTCCAT	CCGAGTACGT	GCTCGCTCGA	TGCGATGTTT
	TGTCAAGCCG	ACCGCGCTCG	GGGACTACGA	GAAGCAGGTC	TAGTAGGACT	AGCTGTTCTG	GCCGAAGGTA	GGCTCATGCA	CGAGCGAGCT	ACGCTACAAA
						neo				
1801	CGCTTGGTGG	TCGAATGGGC	AGGTAGCCGG	ATCAAGCGTA	TGCAGCCGCC	GCATTGCATC	AGCCATGATG	GATACTTTCT	CGGCAGGAGC	AAGGTGAGAT
	GCGAACCACC	AGCTTACCCG	TCCATCGGCC	TAGTTCGCAT	ACGTCGGCGG	CGTAACGTAG	TCGGTACTAC	CTATGAAAGA	GCCGTCCTCG	TTCCACTCTA
						neo				
1901	GACAGGAGAT	CCTGCCCCGG	CACTTCGCCC	AATAGCAGCC	AGTCCCTTCC	CGCTTCAGTG	ACAACGTCGA	GCACAGCTGC	GCAAGGAACG	CCCGTCGTGG
	CTGTCCTCTA	GGACGGGGCC	GTGAAGCGGG	TTATCGTCGG	TCAGGGAAGG	GCGAAGTCAC	TGTTGCAGCT	CGTGTCGACG	CGTTCCTTGC	GGGCAGCACC
						neo				
2001	CCAGCCACGA	TAGCCGCGCT	GCCTCGTCCT	GCAGTTCATT	CAGGGCACCG	GACAGGTCGG	TCTTGACAAA	AAGAACCGGG	CGCCCCTGCG	CTGACAGCCG
	GGTCGGTGCT	ATCGGCGCGA	CGGAGCAGGA	CGTCAAGTAA	GTCCCGTGGC	CTGTCCAGCC	AGAACTGTTT	TTCTTGGCCC	GCGGGGACGC	GACTGTCGGC
						neo				
2101	GAACACGGCG	GCATCAGAGC	AGCCGATTGT	CTGTTGTGCC	CAGTCATAGC	CGAATAGCCT	CTCCACCCAA	GCGGCCGGAG	AACCTGCGTG	CAATCCATCT
	CTTGTGCCGC	CGTAGTCTCG	TCGGCTAACA	GACAACACGG	GTCAGTATCG	GCTTATCGGA	GAGGTGGGTT	CGCCGGCCTC	TTGGACGCAC	GTTAGGTAGA
						neo				
2201	TGTTCAATCA	TGCGAATCGA	TGCTTGGGTA	GAATAGGTAA	GTCAGATTGA	ATCTGAAATA	AAGGGAGGAA	GGGCGAACTT	AAGAAGGTAT	GACCGGGTCG
	ACAAGTTAGT	ACGCTTAGCT	ACGAACCCAT	CTTATCCATT	CAGTCTAACT	TAGACTTTAT	TTCCCTCCTT	CCCGCTTGAA	TTCTTCCATA	CTGGCCCAGC
	neo						TrpC promoter			
2301	TTCACTTACC	TTGCTTGACA	AACGCACCAA	GTTATCGTGC	ACCAAGCAGC	AGATGATAAT	AATGTCCTCG	TTCCTGTCTG	CTAATAAGAG	TCACACTTCG
	AAGTGAATGG	AACGAACTGT	TTGCGTGGTT	CAATAGCACG	TGGTTCGTCG	TCTACTATTA	TTACAGGAGC	AAGGACAGAC	GATTATTCTC	AGTGTGAAGC
						TrpC promoter				
2401	AGCGCCGCCG	CTACTGCTAC	AAGTGGGGCT	GATCTGACCA	GTTGCCTAAA	TGAACCATCT	TGTCAAACGA	CACAAATTTT	GTGCTCACCG	CCTGGACGAC
	TCGCGGCGGC	GATGACGATG	TTCACCCCGA	CTAGACTGGT	CAACGGATTT	ACTTGGTAGA	ACAGTTTGCT	GTGTTTAAAA	CACGAGTGGC	GGACCTGCTG
						TrpC promoter				

2501 TAAACCAAAA TAGGCATTCA TTGTTGACCT CCACTAGCTC CAGCCAAGCC CAAAAAGTGC TCCTTCAATA TCATCTTCTG TCGAAATTCT TGAAGACGAA
ATTTGGTTTT ATCCGTAAGT AACAACTGGA GGTGATCGAG GTCGGTTCGG GTTTTTCACG AGGAAGTTAT AGTAGAAGAC AGCTTTAAGA ACTTCTGCTT

CpGpd terminato

TrpC promoter

AatII

2601 AGGGCCTCGT GATACGCCTA TTTTTATAGG TTAATGTCAT GATAATAATG GTTTCTTAGA CGTCAGGTGG CACTTTTCGG GGAAATGTGC GCGGAACCCC
TCCCGGAGCA CTATGCGGAT AAAAATATCC AATTACAGTA CTATTATTAC CAAAGAATCT GCAGTCCACC GTGAAAAGCC CCTTTACACG CGCCTTGGGG

CpGpd terminator

2701 TATTTGTTTA TTTTTCTAAA TACATTCAAA TATGTATCCG CTCATGAGAC AATAACCCTG ATAAATGCTT CAATAATGGA GCTTGTCGGA TCGTATTCAT
ATAAACAAAT AAAAAGATTT ATGTAAGTTT ATACATAGGC GAGTACTCTG TTATTGGGAC TATTTACGAA GTTATTACCT CGAACAGCCT AGCATAAGTA

CpGpd terminator

2801 TACATTACAT GGTCTGCACT GCTAATTACA GATACCTTTT TTTCTAATTC CTCTTTTCAG AAGCCTGATG AAACTGCGTG CAACGGCTGG CTGCTTCACA
ATGTAATGTA CCAGACGTGA CGATTAATGT CTATGGAAAA AAAGATTAAG GAGAAAAGTC TTCGGACTAC TTTGACGCAC GTTGCCGACC GACGAAGTGT

CpGpd terminator

2901 AGGTGCGAGA ATGTGTGTAT ACACGGATGA ATTTGACCAT CCAGCCAAGT CGCAGGTTTA TGATTTTGAT ATTTGAACGG GTTCACAACA AAATGCACAG
TCCACGCTCT TACACACATA TGTGCCTACT TAAACTGGTA GGTCGGTTCA GCGTCCAAAT ACTAAAACTA TAAACTTGCC CAAGTGTTGT TTTACGTGTC

CpGpd terminator

3001 ATGCCCTGTA GTTCGATGAC TTCGTCTATA GTACTACTAA GGTGGTCCTC ATTTAATCTG ATTTTTTTGT TATTTATTAG GAGTCGCATA CGTAGCTAAC
TACGGGACAT CAAGCTACTG AAGCAGATAT CATGATGATT CCACCAGGAG TAAATTAGAC TAAAAAAACA ATAAATAATC CTCAGCGTAT GCATCGATTG

CpGpd terminator

3101 TACCACTGCA CTAGGCCACA CTCCCCACGG CGCCGACCCA AGCCTTGGTT GTCATGGCCG CAATAGTCGT TGTGACGTTA CCGACTTTTT CTTCGCTGCC
ATGGTGACGT GATCCGGTGT GAGGGGTGCC GCGGCTGGGT TCGGAACCAA CAGTACCGGC GTTATCAGCA ACACTGCAAT GGCTGAAAAA GAAGCGACGG

CpGpd terminator

3201 CCTTTTTAGT TTGTTTTGGT TCATTGCCTT TCCATAATTC CCCCATGGGT TTGTTTCATT ATTGAAAAGC AGGCTTCGCA GTCTCCGAGA CGACGGGAAC
GGAAAAATCA AACAAAACCA AGTAACGGAA AGGTATTAAG GGGGTACCCA AACAAAGTAA TAACTTTTCG TCCGAAGCGT CAGAGGCTCT GCTGCCCTTG

CpGpd terminator

SrfI

SmaI

3301 GGCGCCGTTG ACGCCGTGTT CCAAGGTGTT GGTCGCAGTC TAGGCGTTGC CATCGACCTT GTGCGACGTG GGAGATCAGG TCAAGCATGC CCGGGCCTGC
CCGCGGCAAC TGCGGCACAA GGTTCCACAA CCAGCGTCAG ATCCGCAACG GTAGCTGGAA CACGCTGCAC CCTCTAGTCC AGTTCGTACG GGCCCGGACG

CpGpd terminator

AscI BamHI

3401 AGGCGCGCCC TAGTCATCCT GTACCAATTC CAATCGCAGA CAAGGGTCAT CTTTATCCTT TCTGCGCATT AGCACTTCGT AGTCTGGATC CGTAACCCAA
TCCGCGCGGG ATCAGTAGGA CATGGTTAAG GTTAGCGTCT GTTCCCAGTA GAAATAGGAA AGACGCGTAA TCGTGAAGCA TCAGACCTAG GCATTGGGTT

FgTri1_NX chemotype

-1 * D D Q V L E L R L C P D D K D K R R M L V E Y D P D T V W T

3501 GTGGTGCACC AGGCCATCAG TTGCGGCTCG GGACCTTCAG GAATCTTCCA CTCGTAGTTG AGCAAAAGGT ACATCATGAC AATCTTGACT TGCTTGGCGA
CACCACGTGG TCCGGTAGTC AACGCCGAGC CCTGGAAGTC CTTAGAAGGT GAGCATCAAC TCGTTTTCCA TGTAGTACTG TTAGAACTGA ACGAACCG**CT**

FgTri1_NX chemotype

-1 · TT C W A M L Q P E P G E P I K W E Y N L L L Y M M V I K V Q K A **V**

3601 CAAAGAAGCG ACCAGGGCAG GCGTGTTCTC CGTAACCGAA GCCCATGTGT TCAACACTGG GCGAAGATAG CTGGACCTTG TCTTCTCTGG CGGATTTTTG
GTTTCTTCGC TGGTCCCGTC CGCACAAGAG GCATTGGCTT CGGGTACACA AGTTGTGA**CC** **C**GCTTCTATC GACCTGGAAC AGAAGAGACC GCCT**AAA**AAC

FgTri1_NX chemotype

-1 · F F R G P C A H E G Y G F G M H E V S **P** S S L Q V K D E R A S **K** Q

3701 TCTCTCACGG AAGAATCGGT AACCATCCCA CTTTTCAGGG TTCTCGTAGA TCCTTCCATC GCGCATGTTG TGACAGGAGA CAGCAATTTG ATGACCCTTG
AGAGAGTGCC TTCTTAGCCA TTGGTAGGGT GAAAAGTCCC AAGAGCATCT AGGAAGGTAG CGCGTACAAC ACTGTCCTCT GTCGTTAAAC TACTGGGAAC

FgTri1_NX chemotype

-1 R E R F F R Y G D W K E P N E Y I R G D R M N H C S V A I Q H G K P

3801 GGGATAAATG TACCGTCTGA GAGTGTGATT TCGGACTGCG CTTCTCGCAC CATTGAAGCA ATTTGAAAGG GTTTCAATCG TTGGGTTTCC TTAAATACGC
CCCTATTTAC ATGGCAGACT CTCACACTA**A** **AG**CCTGACGC GAAGAGCGTG GTAACTTCG**T** **TA**AAC**TTT**CC CAAAGTTAGC AACCCAAAGG AATTTATGCG

FgTri1_NX chemotype

-1 · P I F T G D S L T I **E** S Q A E R V M S A **I** Q **F** P K L R Q T E K F V S

BsrGI

3901 TGTCGAGAAG GATCATCTTG TACATAGCCT TCGTGTTCCA CCCTTCTTCG GCAATAACTC TCCTGATCTC TTCCTGCAGG GGCTTGATGA TTTCAGGGTG
ACAGCTCTTC **CTA**GTAGAAC ATGTATCGGA AGCACAAGGT GGGAAGAAGC CGTTATTGAG AGGACTAGAG AAGGACGTCC CCGAACTACT AAAGTCCCAC

FgTri1_NX chemotype

-1 ·S D L L **I** M K Y M A K T N W G E E A I V R R I E E Q L P K I I E P H

4001 AGCGGCAATA TCTTCCAAAC ATTGGCAGAT GAGATCGGTG GTGGTGTGGA CAGATACAGC AGAAAGGAAT AGTTGGGTAG CAGCAGGGTT GTACGCTTGT
TCGCCGTTAT AGAAGGTTTG TAACCGTCTA CTCTAGCCAC CACCACACCT GTCTATGTCG TCTTTCCTTA TCAACCCATC GTCGTCCCAA CATGCGAACA

FgTri1_NX chemotype

-1 A A I D E L C Q C I L D T T T H V S V A S L F L Q T A A P N Y A Q K

4101 TTGGCAGTCT TCTCAAACCA GCCCAGAGAA TCATTGTATT CCTTACCCTT TTCCTCGGAT CGACGGCGTT CAACAATGGG ATTGATCATT TCGCTAGCAT
AACCGTCAGA AGAGTTTGGT CGGGTCTCTT AGTAACATAA GGAATGGGAA AAGGAGCCTA GCTGCCGCAA GTTGTTACCC T**AAC**TA**GTA**A AG**CGA**TCGTA

FgTri1_NX chemotype

-1 ·K A T K E F W G L S D N Y E K G K E E S R R R E V I P **N** I **M** E **S** A N

4201 TCTTGACCTG GTTTCGTAGA ATTCCGCATC CTGGAGAGAA GTATGAAACG ACGTAACGCA TGTACTTGGG CCAGACGCGC AGGCGGTTTG CGACCTCGAG
AGAACTGGAC CAAAGCATCT TAAGGCGTAG GACCTCTCTT CATACTTTGC TGCATTGCGT ACATGAACCC GGTCTGCGCG TCCGCCAAAC GCTGGAGCTC
FgTri1_NX chemotype
-1 · NK V Q N R L I G C G P S F Y S V V Y R M Y K P W V R L R N A V E L

BglII
4301 ACTGTTGGCA GCGTATTCCG TAGTGATGCT AATCCATTTG TCATTTTGGC ATAGATCTTC ACCCAAAAAG ATACGTGTTG AGATTCTAGT GACCAGGGGT
TGACAACCGT CGCATAAG**GC** **A**TCACTACGA TTAGGTAAAC AGTAAAACCG TATCTAGAAG TGGGTTTTTC TATGCACAAC TCTAAGATCA CTGGTCCCCA
FgTri1_NX chemotype
-1 S N A A Y E **T** T I S I W K D N Q C L D E G L F I R T S I R T V L P L

4401 AGCAGTGCGT CTCTGAGTCG AATCTTGTGC CATTCCTTGT TAGCACCGAG GTAGTGTTCA ACAGCCAGCT GGGTTTCTTG AGCCAATGGA AAGGTCATTT
TCGTCACGCA GAGACTCAGC TTAGAACACG GTAAGGAACA ATCGTGGCTC CATCACAAGT TGTCGGTCGA CCCAAAGAAC TCGGTTACCT TTCCAGTAAA
FgTri1_NX chemotype
-1 ·L L A D R L R I K H W E K N A G L Y H E V A L Q T E Q A L P F T M K

4501 TGTTCAGAGT TTTCTTTAAT TGACCGTGAA TAACCTCCTT GATCAGTGCT GCCTCATTTC TTCCGCCCTC GCGGAAAGGC TCAAAACCGG GGATTTCAGT
ACAAGTCTCA AAAGAAATTA ACTGGCACTT ATTGGAGGAA CTAGTCACGA CGGAGTAAAG AAGGCGGGAG CGCCTTTCCG AGTTTTGGCC CCTAAAGTCA
FgTri1_NX chemotype
-1 ·K N L T K K L Q G H I V E K I L A A E N R G G E R F P E F G P I E T

4601 GTGCAAGGCC GAATCGTTAG CTGCCTTGAT GAAGCTGAGG CGCTTATCGT TCTTGATTTC ATCGGCCAGA GTGGGAGGCA AAAAGGTCAT TTCACCGAAA
CAC**GTT**CCGG CTTAGCAATC GACGGAACTA CTTCGACTCC GCGAATAG**CA** **A**GAACTAAAG TAGCCGGTCT CACCCTCCGT TTTTCCAGTA AAGTGGCTTT
FgTri1_NX chemotype
-1 H **L** A S D N A A K I F S L R K D **N** K I E D A L T P P L F T M E G F D

4701 TCGGTTGTCA GGCGGACAGG GGTTGTTGGG TGCTTGTGGA ACCATTCTTC GAGGGTTTGG CGGGTGGTGT GAAGGACTTC TGAGACTGCA CGAGCATCGG
AGCCAACAGT CCGCCTGTCC CCAACAACCC ACGAACACCT TGGTAAGAAG CTCCCAAACC GCCCACCACA CTTCCTGAAG ACTCTGACGT GCTCGTAGCC
FgTri1_NX chemotype
-1 · DT T L R V P T T P H K H F W E E L T Q R T T H L V E S V A R A D T

4801 TAAACTCGTA CCAGCGTCTG GGGTTGAGCA AGTATGCGCT GGGTTGCGGG GTCAGCTTCT TTCGTACGAC GGATACTACG ACGAGAAAGG CAACAAAATA
ATTTGAGCAT GGTCGCAGAC CCCAACTCGT TCATACGCGA CCCAACGCC**C** **CA**GTCGAAGA AAGCATGCTG CCTATGATGC TGCTCTTTCC GTTGTTTTAT
FgTri1_NX chemotype
-1 · TF E Y W R R P N L L Y A S P Q P **T** L K K R V V S V V V L F A V F Y

PacI
HindIII
4901 TGCCAGCATG TCGAGCCGGA CATCCTGCAA ACTGGTGATG AGAGCCATTT AATTAAGCTT GATTGGAATT CGGTGAAGGT TGTGTTATGT TTTGTGGAAT
ACGGTCGTAC AGCTCGGCCT GTAGGACGTT TGACCACTAC TCTCGGTAAA TTAATTCGAA CTAACCTTAA GCCACTTCCA ACACAATACA AAACACCTTA
FgTri1_NX chemotype ← *FgTri1_NX chemotype* AnTef_promoter
-1 A L M D L R V D Q L S T I L A M

5001 ATAAAAGGGA GAAGTGCTCA GATACTACGG CTAGTAATGC GTAGAGGTGC TGAGCAGAGA AGTTAGCAGG AGATGGATGA AAGAAGGATG GAGGAGAGAA
TATTTTCCCT CTTCACGAGT CTATGATGCC GATCATTACG CATCTCCACG ACTCGTCTCT TCAATCGTCC TCTACCTACT TTCTTCCTAC CTCCTCTCTT
AnTef_promoter

5101 GCGAGGGAAG GGAAGATATA CGTACTGAAG CGGACAAGGA GAGGAAGGGA AAGAAAATTT TGTGGGAGGG GGAGGGGAAA TCCTTTTATG GAGGGGCAAG
CGCTCCCTTC CCTTCTATAT GCATGACTTC GCCTGTTCCT CTCCTTCCCT TTCTTTTAAA ACACCCTCCC CCTCCCCTTT AGGAAAATAC CTCCCCGTTC
AnTef_promoter

EcoRV
5201 CGAGGACGAC GACTAAGCGG GCGGGCTCAA ACCCTTGGCT GGTAGATATC TGTCATTGGA TATGGAGGGG TAACCCGCCC CACTCCCGAT GCAAATTCTT
GCTCCTGCTG CTGATTCGCC CGCCCGAGTT TGGGAACCGA CCATCTATAG ACAGTAACCT ATACCTCCCC ATTGGGCGGG GTGAGGGCTA CGTTTAAGAA
AnTef_promoter

5301 CTCCTAGACT CCCACCTTGG GCGTCGAGCC TTTACAACAC CTTCCGGAAC GGCAGCCCAG CGCGACTCTT TCCTTGGTTC TGCTCCACCC CATCTCGTTT
GAGGATCTGA GGGTGGAACC CGCAGCTCGG AAATGTTGTG GAAGGCCTTG CCGTCGGGTC GCGCTGAGAA AGGAACCAAG ACGAGGTGGG GTAGAGCAAA
AnTef promoter

5401 TCATGGCTTA GGGGCAGAAA TTTTTTTTTG TTTAGCTTTT CTTTACTTGC TATCGCCTCT ACGGTGGATT CTCGGATCGA AAAAGCTGGG CATCAACGCG
AGTACCGAAT CCCCGTCTTT AAAAAAAAAC AAATCGAAAA GAAATGAACG ATAGCGGAGA TGCCACCTAA GAGCCTAGCT TTTTCGACCC GTAGTTGCGC
AnTef_promoter

5501 TTGATGGTCG CATGGCGGCT TTTATCACTC TGTACTTTGC AGCGGACAAC CCCGCGGCTG TACGAGTTTG CCGCCTTTTA ATTTGTCTGC GACCAGCCTG
AACTACCAGC GTACCGCCGA AAATAGTGAG ACATGAAACG TCGCCTGTTG GGGCGCCGAC ATGCTCAAAC GGCGGAAAAT TAAACAGACG CTGGTCGGAC
AnTef promoter

5601 CGATGGAGCG AGCGAGTGGC GTTGCTTGAT CCACAGCGTC AAGATCAACA TACGTAGCAA GATAGACCGC AAGAAAATGA TGCTCGGAAT CCATATTGAC
GCTACCTCGC TCGCTCACCG CAACGAACTA GGTGTCGCAG TTCTAGTTGT ATGCATCGTT CTATCTGGCG TTCTTTTACT ACGAGCCTTA GGTATAACTG
AnTef_promoter

5701 TCACGAAAAT TTCTTTCTTT TTGCCCCCCT CATCTGTTGC CTGGGAGGTG AGTCAAATTG CCCCTCACTA AACGCTCCTT TGCTTTTCCC GAACTTGGCC
AGTGCTTTTA AAGAAAGAAA AACGGGGGGA GTAGACAACG GACCCTCCAC TCAGTTTAAC GGGGAGTGAT TTGCGAGGAA ACGAAAAGGG CTTGAACCGG
AnTef promoter

5801 CGTTCGAGAG CATGATCAGC ACAAAGGCTT AACTTGGGCG GTGATTCTGC TGTCTCGTAT TAATAAACTA TAATAATTCT GTTTTTTTTT TTATGCTTCA
GCAAGCTCTC GTACTAGTCG TGTTTCCGAA TTGAACCCGC CACTAAGACG ACAGAGCATA ATTATTTGAT ATTATTAAGA CAAAAAAAAA AATACGAAGT
AnTef_promoter

NotI Sac
5901 GGCGTACTAT ATGCCATATA CAAATGCTAT CAAATCAGAA GTTAGGGGTA TCTTTTGAGC GTGCACTCTA AGTCGCTAGC GGCCGCCACC GCGGTGGAGC
CCGCATGATA TACGGTATAT GTTTACGATA GTTTAGTCTT CAATCCCCAT AGAAAACTCG CACGTGAGAT TCAGCGATCG CCGGCGGTGG CGCCACCTCG
AnTef_promoter

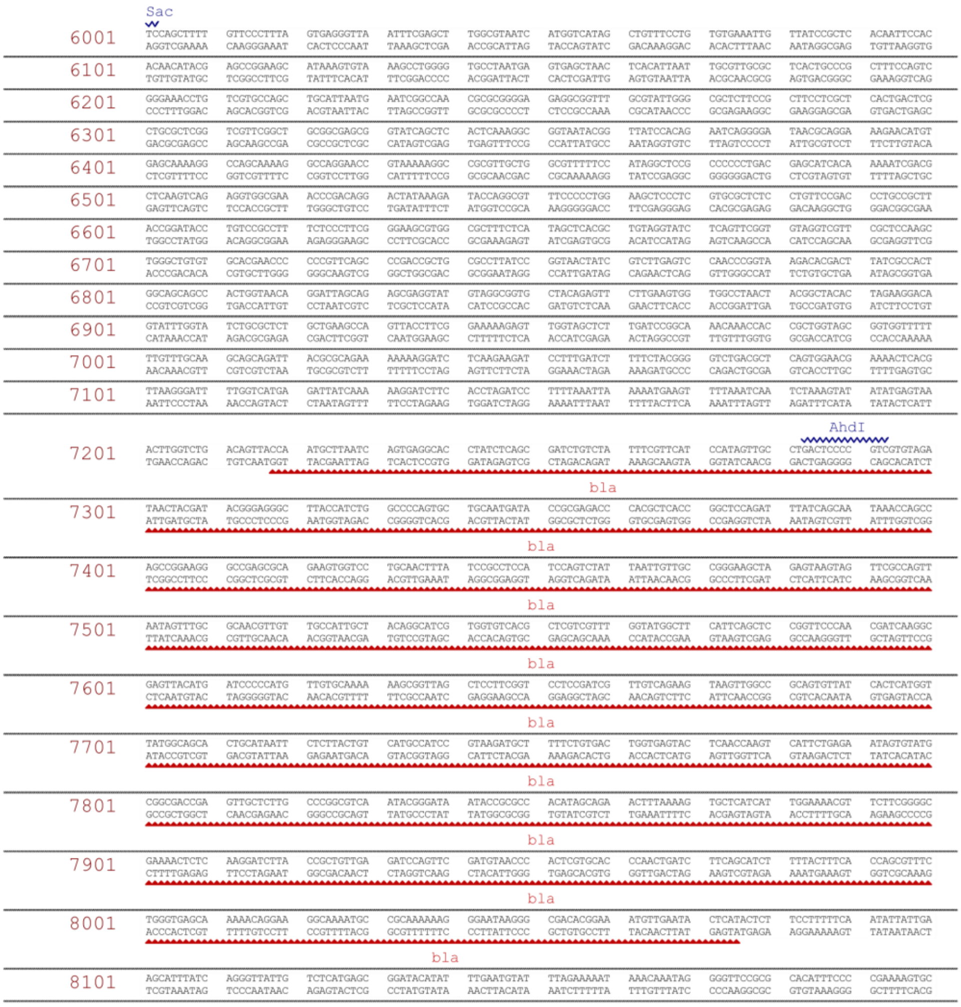

Figure S2. The C-7 hydroxylase overexpression vector, pAnTef-*FgTri1*_*NX chemotype*-neo. Modified bases on *FgTri1* sequence are listed below the map.

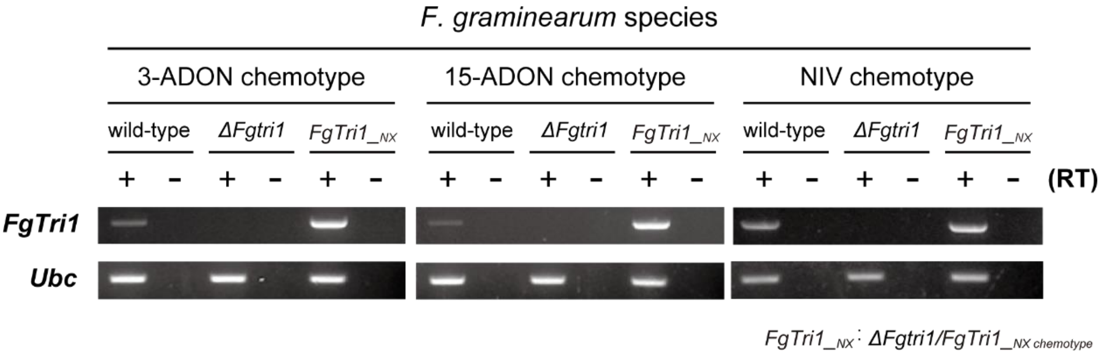

Figure S3. PCR analysis of the *FgTri1*_*NX chemotype* expression in transgenic strains. Mycelia cultured for 3 days in trichothecene induction medium were used. RT: reverse transcription; Ubc: ubiquitin-conjugating enzyme.
